# DeepSuccinylSite: a deep learning based approach for protein succinylation site prediction

**DOI:** 10.1186/s12859-020-3342-z

**Published:** 2020-04-23

**Authors:** Niraj Thapa, Meenal Chaudhari, Sean McManus, Kaushik Roy, Robert H. Newman, Hiroto Saigo, Dukka B. KC

**Affiliations:** 1 Department of Computational Science and Engineering, North Carolina A&T State University, Greensboro, NC USA; 2 Department of Computer Science, North Carolina A&T State University, Greensboro, NC USA; 3 Department of Biology, North Carolina A&T State University, Greensboro, NC USA; 4 Faculty of Information Science and Electrical Engineering, Kyushu University, Fukuoka, Japan; 5 Electrical Engineering and Computer Science Department, Wichita State University, Wichita, KS USA

**Keywords:** Succinylation, Deep learning, Convolutional neural network, Recurrent neural network, Long short-term memory, Embedding

## Abstract

**Background:**

Protein succinylation has recently emerged as an important and common post-translation modification (PTM) that occurs on lysine residues. Succinylation is notable both in its size (e.g., at 100 Da, it is one of the larger chemical PTMs) and in its ability to modify the net charge of the modified lysine residue from + 1 to − 1 at physiological pH. The gross local changes that occur in proteins upon succinylation have been shown to correspond with changes in gene activity and to be perturbed by defects in the citric acid cycle. These observations, together with the fact that succinate is generated as a metabolic intermediate during cellular respiration, have led to suggestions that protein succinylation may play a role in the interaction between cellular metabolism and important cellular functions. For instance, succinylation likely represents an important aspect of genomic regulation and repair and may have important consequences in the etiology of a number of disease states. In this study, we developed DeepSuccinylSite, a novel prediction tool that uses deep learning methodology along with embedding to identify succinylation sites in proteins based on their primary structure.

**Results:**

Using an independent test set of experimentally identified succinylation sites, our method achieved efficiency scores of 79%, 68.7% and 0.48 for sensitivity, specificity and MCC respectively, with an area under the receiver operator characteristic (ROC) curve of 0.8. In side-by-side comparisons with previously described succinylation predictors, DeepSuccinylSite represents a significant improvement in overall accuracy for prediction of succinylation sites.

**Conclusion:**

Together, these results suggest that our method represents a robust and complementary technique for advanced exploration of protein succinylation.

## Background

Protein post-translational modifications (PTM) are important cellular regulatory processes that occur after protein synthesis. PTMs increase the functional diversity of the proteome by the covalent addition of functional moieties to proteins, proteolytic cleavage of regulatory subunits and play important roles in signaling for degradation of entire proteins. PTMs include phosphorylation, glycosylation, ubiquitination and relatively recently described modifications, such as succinylation. Succinylation is a PTM that occurs through the addition of a succinyl group (−CO-CH_2_-CH_2_-CO_2_H) to the ε-amino of target lysine residues.

Protein PTMs have been detected by a variety of experimental techniques [1], including mass spectrometry (MS) [2, 3], liquid chromatography [4], radioactive chemical labeling [5] and immunological detection, such as chromatin immunoprecipitation [6] and western blotting [7]. Generally, the experimental analysis of PTMs requires time-consuming, labor- and capital-intensive techniques and the use of hazardous/expensive chemical reagents. Due to importance of PTMs in both disease states and normal biological functions, it is imperative to invest in developing options that can screen proteins for potential PTM sites in a rapid, cost-effective manner.

In recent years, machine learning has become a cost-effective method for prediction of different PTM sites. Some of the machine learning based succinylation site prediction approaches are iSuc-PseAAC [8], iSuc-PseOpt [9], pSuc-Lys [10], SuccineSite [11], SuccineSite2.0 [12], GPSuc [13] and PSuccE [14] . Although results have been promising, the potential for bias is present due to manual selection of features along with the possible absence of unknown features that contribute to succinylation. Moreover, the prediction performance of these methods is not yet satisfactory enough to be used in high throughput studies.

Recently, deep learning (DL) approaches have been developed to elucidate putative PTM sites in cellular proteins. For instance, MusiteDeep [15] and DeepPhos [16] have been developed to predict phosphorylation sites while Fu et al. [17] and Wu et al. [18] used DL-based methods to identify putative ubiquitination and acetylation sites, respectively. These DL methods have achieved relative improvement in aggregate measures of method performance, such as the area under curve (AUC) and Matthews Correlation Coefficient (MCC). Typically, these models utilize some combination of one-hot encoding and extracted features as an input, largely trying to avoid reliance on manual feature extraction. To the best of our knowledge, DL models have not been applied previously for prediction of succinylation sites. In this study, we developed a succinylation site predictor, termed DeepSuccinylSite, based on a convolutional neural network (CNN) deep learning framework [19] using Keras library [20].

## Methods

### Benchmark dataset

In this study, we used the same training and independent dataset collected from experimentally derived lysine succinylation sites as in Hasan et al. [13] and Ning et al. [14]. Ning et al. used UniProtKB/Swiss-Prot database and NCBI protein sequence database as Hasan et al. to create the succinylation dataset. After removing proteins that have more than 30% sequence identity using CD-HIT, 5009 succinylation sites and 53,542 sites not known to be succinylated remained. Of these, 4755 succinylation sites and 50,565 non-succinylation sites were used for the training set and 254 succinylation sites and 2977 non-succinylation sites were used for the independent test. Moreover, for our approach the optimal window size came out to be 33 and some of the sequences had other characters, we lost 5 (out of 4755) positive sites in the training set.

For the training and test sets, data were balanced using under-sampling. The final training dataset contained 4750 positive and 4750 negative sites whereas the independent test dataset contained 254 positive and 254 negative sites after balancing. Table 1 shows the final dataset for training and independent test after balancing. In order to generate a local representation of the protein and to optimize the model, a window parameter was set around each lysine (K) of interest. If the left or right side of K was less than half the size of the window, then pseudo residue “-” was used in order to retain all the positive sites.

**Table 1:** Number of positive and negative sites for training and testing dataset

Dataset	Positive	Negative
Training	4750	4750
Independent Test	254	254

### Encoding

In contrast to traditional machine learning methods, our DL-based method takes sequence data in the form of windows directly as an input, reducing the need for hand-crafted feature extraction. A pre-requisite for this approach is that the sequence data must be encoded in a form that is readable by our DL model. Accordingly, we have utilized two types of encoding: (i) one-hot encoding and (ii) embedding layer. Compared to other DL approaches for other types of post-translational modification site prediction, one of the major differences is our embedding encoding.

#### One-hot encoding

One hot encoding converts categorical variables to respective binary variables. We implemented one-hot encoding in a manner similar to that used during the development of MusiteDeep [15]. In order to convert the 20 common amino acids and our pseudo residue “-” into numerical values, these 21 characters are converted into integers ranging from 0 to 20. Every amino acid was represented by a binary code consisting of a sequence of zeros and a singular one, the location of which encodes the identity of the amino acid. In our study, the binary representation was done based on alphabetical order. For example, Alanine (A) is represented as 100000000000000000000 and Arginine (R) is represented as 010000000000000000000 and so on. Accordingly, in our model, a window of size, N, corresponded to an input vector size of N × 21.

One of the primary drawbacks of one-hot encoding is that the mapping is completely uniform. Therefore, amino acids with similar properties are not placed together in vector space.

#### Embedding layer

One of the highlights of our approach is the embedding layer. The second type of encoding that we utilize is the embedding encoding [20, 21]. Embedding finds the best representation for the amino acid sequence, as in DeepGO [22], to overcome the shortcomings of one-hot encoding. Briefly, the 20 amino acids residue and 1 pseudo residue were first converted into integers ranging from 0 to 20. This is provided as an input to the embedding layer, which lies at the beginning of our DL architecture. The embedding layer is initialized with random weights. The layer then learns better vector-based representations with subsequent epochs during training. Each vectorization is an orthogonal representation in another dimension, thus preserving its identity. Hence, making it more dynamic than the static one-hot encoding. In our study, embedding encoding (word to vec) for K is: [− 0.03372079, 0.01156038, − 0.00370798, 0.00726882, − 0.00323456, − 0.00622324, 0.01516087, 0.02321764, 0.00389882, − 0.01039953, − 0.02650939, 0.01174229, − 0.0204078, − 0.06951248, − 0.01470334, − 0.03336572, 0.01336034, − 0.00045607, 0.01492316, 0.02321628, − 0.02551141] in 21-dimensional vector space after training. Embedding groups commonly co-occurring items together in the vector space. Two key arguments must be specified in the embedding layer. These are:

- output_dim: Size of vector space.
- input_length: Size of input, which is window size.

### Training and testing datasets

The training dataset was further sub-divided into 80% training and 20% validation sets. The model was trained on 80% of the training data with validation done in every epoch using the remaining 20% of the training dataset. This validation approach was performed in order to track the training progress and to identify overfitting. Overfitting was identified when validation accuracy started decreasing while training accuracy continued to increase. Checkpointer was utilized to select the optimal model from the epochs based on validation accuracy; this approach also helped to minimize any potential overfitting. The model generated was then used for independent testing with the independent testing dataset.

### Input

The main advantage of using DL over traditional machine learning approaches is the exclusion of manual feature extraction. The input for our DL approach is the sequence windows in FASTA format. For example, for a window size of 33, the input dimension would be 33 × 21 for one-hot encoding. For embedding for the same window size, the input dimension would be 33 × 21 for embedding output dimension of 21.

### DeepSuccinylSite architecture

**Fig. 1:**
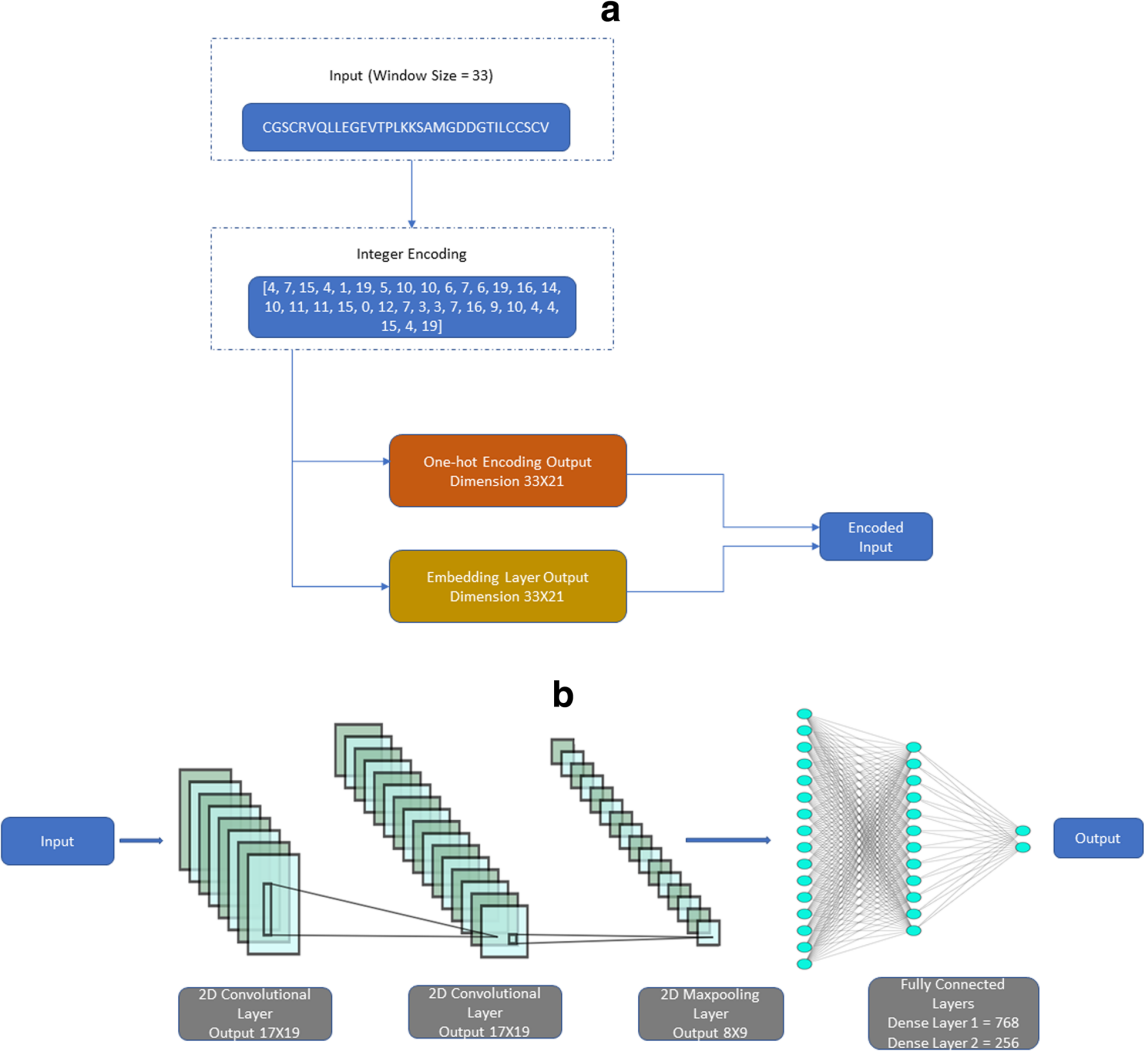
**a** Window size of 33 in FASTA format is the input. It is converted into integers which is then encoded either using one-hot encoding or embedding layer. This will be the input for CNN layers. **b** The output from either of the encoding is then fed as input into the deep learning architecture. Finally, after the flattening and fully connected layers we get the final output which contains two nodes with outputs [0 1] for positive and [1 0] for negative sites

The overall architecture of DeepSuccinylSite is shown in Fig. 1.

After encoding the input data, the encoded data was fed into the network. The same architecture was utilized for both encoding methods, except for the inclusion of an embedding layer and a lambda layer in the case of the embedding encoding.

The next layer is the convolutional layer. Previous DL-based models for Phosphorylation sites (DeepPhos, MusiteDeep) [19, 20] have used 1-D (dimensional) convolutional layer, whereas we have used 2-D (dimensional) convolutional layer, thus increasing our flexibility with choosing 2-D size. If we use 1D convolutional layer and do the same, then we will not be able to deduce many feature information, as the x-axis is fixed (it will stay at 21) and will only stride vertically. Thereafter, other layers were also chosen with 2D. We used a 2D convolutional layer to prioritize the inclusion of filter size 17 × 3 (for window size 33, the PTM site lies at the 17th position), which will include the PTM site in every stride. The use of this filter size, along with the disabling of padding, allowed the model to be optimized for training time without compromising performance. Higher dropout of 0.6 was used to avoid overfitting. Moreover, a rectified linear unit (ReLU) was used as an activation function for all layers. ReLU was deemed an optimal activation function due to its sparse activation, which minimized the possibility for overfitting and maximized the predictive power of the model. We used two convolutional layers, one maxpooling layer, a fully connected layer with two dense layers, and an output layer. The parameters used in the model are given in Table 2.

**Table 2:** Parameters in DeepSuccinylSite

Parameters	Settings
Embedding Output Dimension	21
Learning Rate	0.001
Batch Size	256
Epochs	80
Conv2d_1 number of filters	64
Conv2d_1 filter size	17 × 3 (For window size 33)
Conv2d_1 padding	Disabled
Dropout	0.6
Conv2d_1 number of filters	128
Conv2d_1 filter size	3 × 3
Conv2d_1 padding	Enabled
Dropout	0.6
MaxPooling2d	2 × 2
Dense 1	768
Dropout	0.5
Dense_2	256
Dropout	0.5
Checkpointer	Best validation accuracy

Adam optimization was used as the optimizer for our architecture, as described previously by Kingma et al. [23]. Adam uses an adaptive learning rates methodology to calculate individual learning rates for each parameter. Adam is different from classical stochastic gradient descent in that stochastic gradient descent maintains a single, constant learning rate for all weight updates during training [24]. Specifically, Adam combines benefits of both adaptive gradient algorithm and root mean square propagation, allowing for efficient training of the model. Since this study is a binary classification problem, binary cross-entropy (measure of uncertainty associated with given distribution) or log loss was used as the loss function. The binary cross-entropy is given by:

1 $$ -\frac{1}{N}\sum \limits_{i=1}^N\left[{y}_i\mathit{\log}\left({\hat{y}}_i\right)+\left(1-{y}_i\right)\mathit{\log}\left(1-{\hat{y}}_i\right)\right] $$

where y is the label **(**1 for positive and 0 for negative) and ${\hat{y}}_i$ is the predicted probability of the site being positive for all N points. For each positive site (*y = 1*), it adds $\log \left({\hat{y}}_i\right)$ to the loss, that is, the log probability of it being positive**.** Conversely, for each negative site (*y = 0*), it adds $\log \left(1-{\hat{y}}_i\right)$, that is, the log probability of it being negative.

The fully connected layers contained two dense layers with 768 and 256 nodes, respectively, with the final output layer containing 2 nodes.

### Model evaluation and performance metrics

In this study, 10-fold cross validation was used to evaluate the performance of the model. In 10-fold cross validation, the data are partitioned into 10 equal parts. Then, one-part is left out for validation and training is performed on remaining 9 parts. This process is repeated until all parts are used for validation.

Confusion Matrix (CM), Matthew’s Correlation Coefficient (MCC) and Receiver Operating Characteristics (ROC) curve were used as performance metrics. The ROC curve is a graphical plot that illustrates the diagnostic ability of a binary classifier whereas area under curve (AUC) represents the degree or measure of separability. Since identification of succinylation sites is a binary classification problem, the confusion matrix size is 2 × 2 composed of true positives (TP), true negatives (TN), false positives (FP) and false negatives (FN). Other metrics calculated using these variables were accuracy, sensitivity (i.e., the true positive rate) and specificity (i.e., the true negative rate).

2 $$ Accuracy=\frac{TP+ TN}{TP+ TN+ FP+ FN}\times 100 $$

3 $$ Sensitivity=\frac{TP}{TP+ FN}\times 100 $$

4 $$ Specificity=\frac{TN}{TN+ FP}\times 100 $$

5 $$ MCC=\frac{(TP)(TN)-(FP)(FN)}{\sqrt{\left( TP+ FP\right)\left( TP+ FN\right)\left( TN+ FP\right)\left( TN+ FN\right)}} $$

## Results

### Optimal window size and encoding

**Fig. 2:**
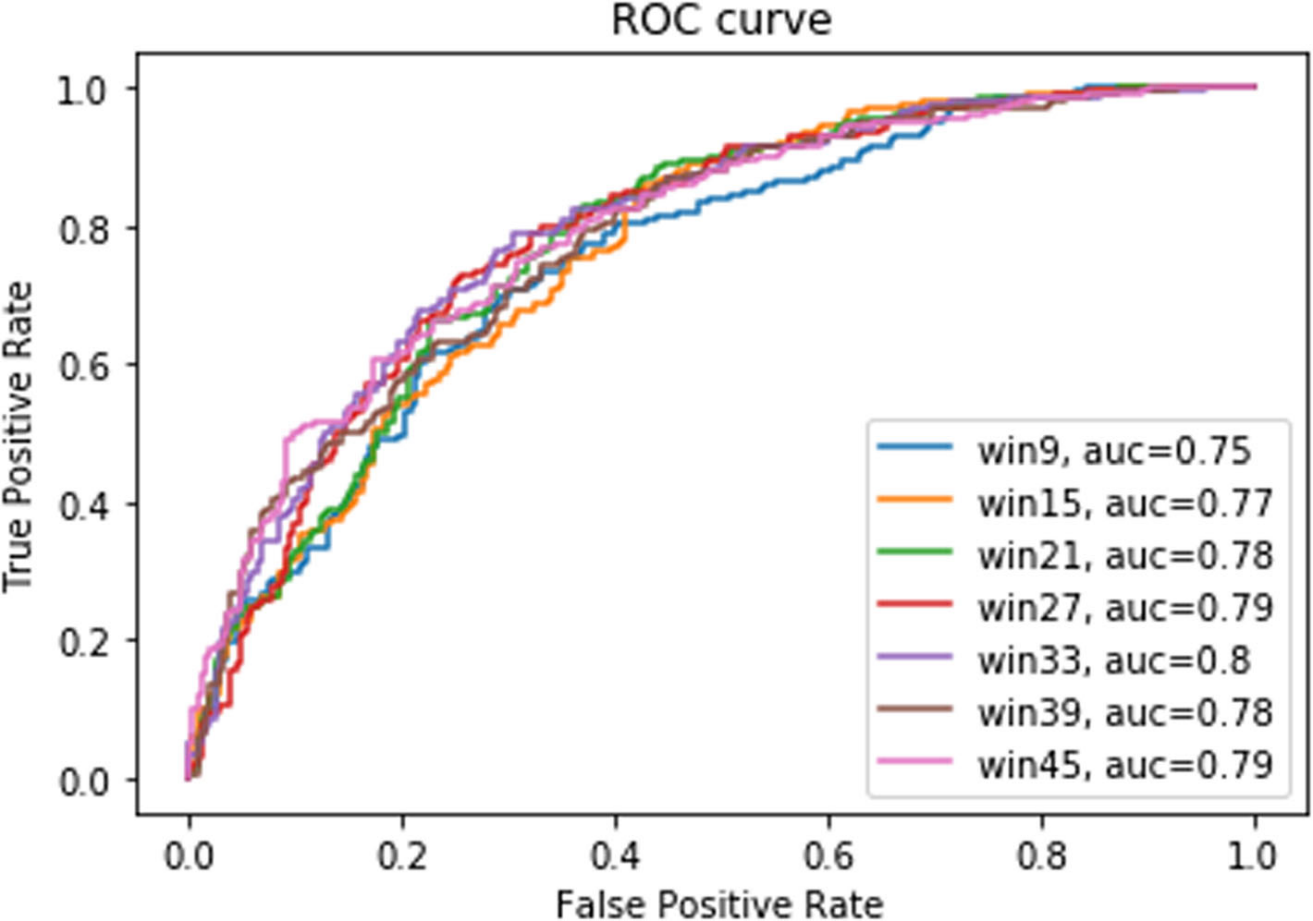
ROC curve for different window sizes for embedding

Initially, window sizes from 9 to 45 were tested with both one-hot encoding and embedding. For example, for a window size of 9, the lysine (K) residue was set in the middle of the window with 4 amino acid residues upstream and 4 amino acid residues downstream. A window size of 33 yielded the highest MCC for both one-hot encoding and embedding, with further increases in window size resulting in reductions in MCC (Table 3). Likewise, the highest specificity and AUC were achieved using a window size of 33, with only a marginal reduction in sensitivity when using embedding (Table 3 and Fig. 2). Hence, a window size of 33 was considered as the optimal window size for this study. Interestingly, a window size of 33 was also utilized by Wang et al. for phosphorylation site prediction using one-hot encoding [15]. It is worth noting that the consistency in window size between this study and the previous study by Wang et al. correlates with the known range for many inter-protein amino acid interactions. Importantly, with only a few exceptions, embedding performed better than one-hot encoding for every window size tested. Therefore, for this study, embedding was chosen for encoding.

**Table 3:** Performance metrics for different window sizes. The highest values in each category are highlighted in boldface. MCC: Matthew’s Correlation Coefficient

Window Size	One-Hot Encoding			Embedding (Dimension = 21)		
	Sensitivity	Specificity	MCC	Sensitivity	Specificity	MCC
9	0.70	0.55	0.25	0.80	0.57	0.39
15	0.73	**0.60**	0.33	**0.82**	0.58	0.42
21	0.79	0.55	0.34	0.76	0.67	0.43
27	0.79	0.59	0.38	0.81	0.63	0.45
33	**0.84**	0.55	**0.41**	0.79	**0.69**	**0.48**
39	0.81	0.53	0.36	0.75	0.63	0.40
45	0.81	0.55	0.38	0.76	0.67	0.43

### Identification of optimal embedding dimension

**Fig. 3:**
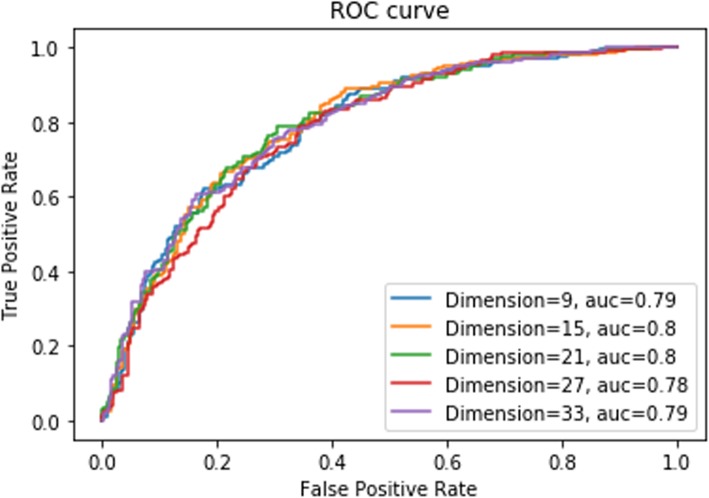
ROC curves for different embedding dimensions

Next, we sought to identify the optimal embedding dimension. To this end, dimensions ranging from 9 to 33 were tested for embedding. It is important to note that increasing the dimension of embedding will result in higher computational cost. Therefore, we aimed to identify the smallest dimension that struck a balance across all metrics. Because MCC is often used as a surrogate of overall model performance, it was prioritized slightly over the other parameters. While both dimension sizes of 15 and 21 struck such a balance, the performance metrics were generally better using a dimension size of 21. Indeed, a dimension size of 21 achieved the highest MCC, with sensitivity and specificity scores that were within 7% of the maximum scores achieved in these areas (Table 4). Consistently, dimension size of 15 and 21 achieved the highest AUC score (Fig. 3). Taken together, these data suggest that a dimension size of 21 is optimal using our architecture. Therefore, a dimension size of 21 was selected for model development. The dimension size is consistent with the fact that 20 amino acid residues and 1 pseudo residue were present in each vector.

**Table 4:** Performance metrics for different embedding dimensions. The highest values in each category are shown in bold. MCC: Matthew’s Correlation Coefficient

Dimension	Sensitivity	Specificity	MCC
9	**0.85**	0.58	0.45
15	0.73	**0.71**	0.44
21	0.79	0.67	**0.48**
27	0.75	0.66	0.41
33	0.77	0.68	0.45

### Cross-validation and alternative classifiers

Our final model, which we termed DeepSuccinylSite, utilizes embedding with window and dimension sizes of 33 and 21, respectively. Based on five rounds of 10-fold cross-validation, DeepSuccinylSite exhibited robustness with consistent performance metrics with an average MCC of 0.519 +/− 0.023 and an AUC of 0.823 (Additional file 1: Table S3). We also implemented additional Deep Learning architectures and different machine learning models where the input was hand-crafted ‘physico-chemical’ based features rather than the protein sequence alone. Essentially, this implementation takes various physiochemical features combined with XGBoost to extract prominent features. We excluded any sequences with ‘-‘, while calculating the features. We then used XGBoost to extract prominent features, which provided better accuracy and obtained a total of 160 features at threshold of 0.00145. Interestingly, the performance of the methods using these approaches were not as good as DeepSuccinylSite, whose input is protein sequence alone (Additional file 1: Table S2). Further information on performance of our model are included in Additional file 1. Additionally, the results of feature-based Deep Learning architecture is shown in Additional file 1: Figure S1.

### Comparison with other deep learning architectures

Other DL architectures, such as Recurrent Neural Network (RNN) [25] and Long Short-Term Memory (LSTM) [26], as well as the combined model, LSTM-RNN, were also implemented for one-hot encoding (DeepSuccinylSite-one_hot) and compared with the independent test result of DeepSuccinylSite (Table 5). Additionally, we implemented an additional DL architecture, where the input includes other features beyond the primary amino acid sequence. Specifically, this implementation utilizes a combination of 1) physiochemical features, such as Pseudo Amino acid Composition (PAAC), ‘k-Spaced Amino Acid Pairs’ (AAP); 2) Autocorrelation features, such as Moreau-Broto autocorrelation and Composition, Transition and Distribution (CTD) features, and 3) Entropy Features, such as Shannon entropy, Relative entropy, and Information gain. We excluded any sequences with ‘-‘, while calculating the features. We then used XGBoost to extract prominent features which provided better accuracy and obtained a total of 160 features at threshold 0.00145. The version of the algorithm using features is termed as DeepSuccinylSite-feature based.

**Table 5:** Comparison of DeepSuccinylSite with other deep learning architectures for window size 33. The highest value in each category is shown in bold. MCC: Matthew’s Correlation Coefficient; RNN: Recurrent neural network; LSTM: Long short-term memory model

Models	Sensitivity	Specificity	MCC
RNN	0.70	0.49	0.20
LSTM-RNN	0.66	0.57	0.23
LSTM	0.74	0.66	0.36
*DeepSuccinylSite-feature based*	*0.80*	*0.44*	*0.27*
DeepSuccinylSite-one_hot	**0.84**	0.55	0.41
DeepSuccinylSite-Embedding	0.79	**0.69**	**0.48**

**Fig. 4:**
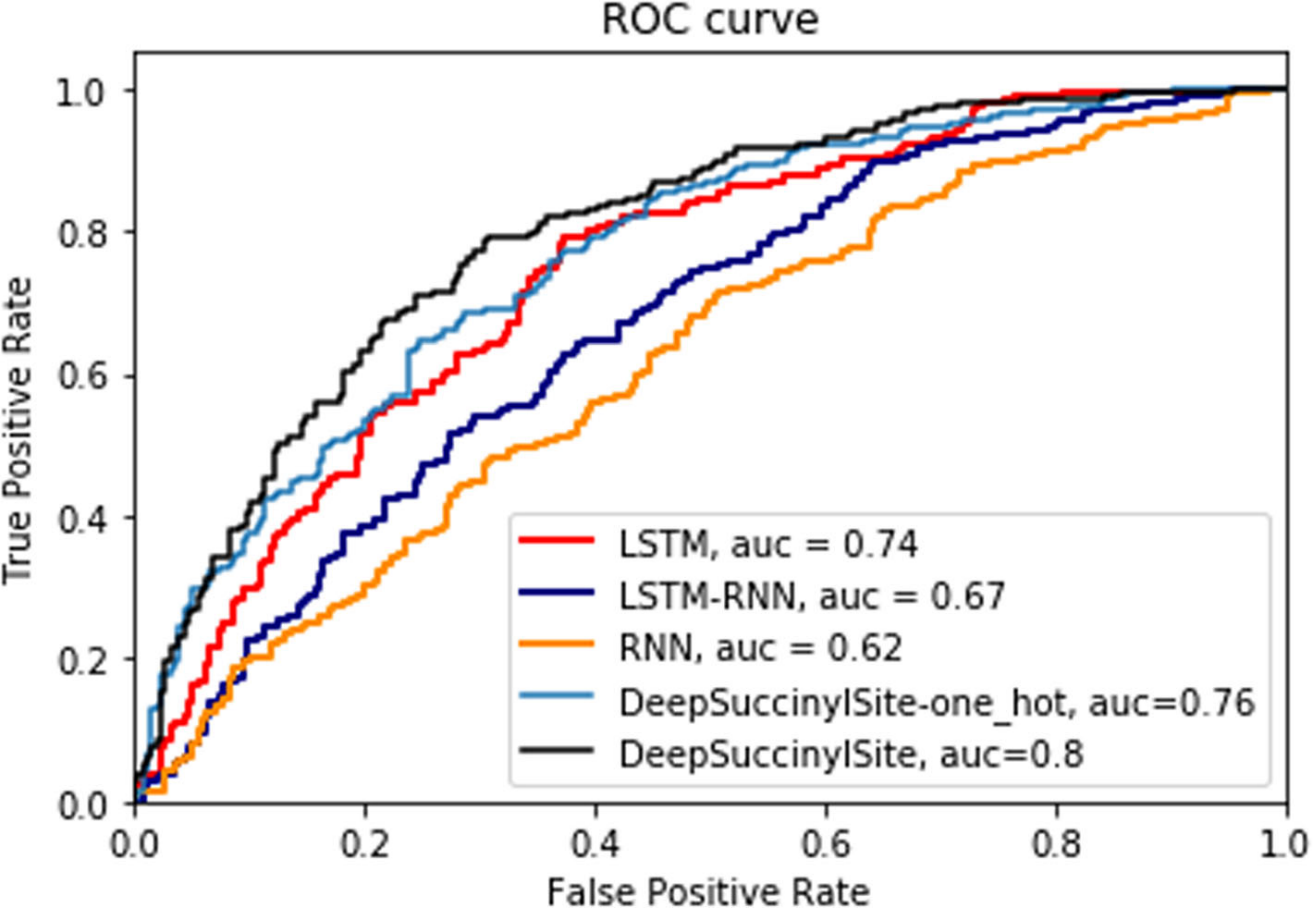
ROC curve for different deep learning architectures

For fair comparison, we used the same balanced training and testing dataset for window size of 33 and one-hot encoding for these three DL architectures. The results are shown in Table 5 and ROC curve is shown in Fig. 4. The results for our DL model with embedding (DeepSuccinylSite) is also shown. The detailed architecture of these models, including results for other window sizes are discussed in Additional file 1 and the performance of these methods is presended in Additional file 1: Table S1. For one-hot encoding, DeepSuccinylSite achieved better MCC and AUC score than the other DL architectures. Likewise, our final model using embedding achieved the highest MCC and AUC scores of any model (Table 5).

### Independent test comparisons with existing models

Next, the performance of DeepSuccinylSite was compared with other succinylation site predictors using an independent test set as mentioned in the benchmark dataset earlier. During these analyses, some of the most widely used tools for succinylation site prediction, such as iSuc-PseAAC [8], iSuc-PseOpt [9], pSuc-Lys [10], SuccineSite [11], SuccineSite2.0 [12], GPSuc [13] and PSuccE [14], were considered. All these methods use the same training and independent test data sets as in Table 6. The performance metrics for these previously published methods were taken from their respective manuscripts mainly based on comparison done in PSuccE [14].

**Table 6:** Comparison of DeepSuccinylSite with existing predictors using an independent test dataset. The highest value in each category is shown in bold

Prediction Schemes	Sensitivity	Specificity	MCC
iSuc-PseAAC	0.12	**0.89**	0.01
iSuc-PseOpt	0.30	0.76	0.04
pSuc-Lys	0.22	0.83	0.04
SuccineSite	0.37	0.88	0.20
SuccineSite2.0	0.45	0.88	0.26
GPSuc	0.50	0.88	0.30
PSuccE	0.38	**0.89**	0.20
DeepSuccinylSite	**0.79**	0.69	**0.48**

DeepSuccinylSite achieved a 58.3% higher sensitivity score than the next highest performing model (Table 6). In contrast, our model exhibited the lowest specificity score of all of models tested. However, the specificity score achieved by DeepSuccinylSite was only 22.2% lower than that of the top-ranked methods. Consequently, DeepSuccinylSite achieved a significantly higher performance as measured by MCC. Indeed, DeepSuccinylSite exhibited an ~ 62% increase in MCC when compared to the next highest method, GPSuc. Taken together, the novel architecture we have described, termed DeepSuccinylSite, shows significantly improved performance for precise and accurate prediction of succinylation sites.

## Discussion

Succinylation is relatively newly discovered PTM that is garnering interest due to the biological implications of introducing a large (100 Da) chemical moiety that changes the charge of the modified residue. Experimental detection of succinylation is labor intensive and expensive. Due to the availability of a relatively large dataset containing 4750 positive sites for training, it was possible for us to implement different DL architectures. The model optimization process described in this paper led to a significant improvement in precise prediction of succinylation sites when compared to models previously described in the literature. Two types of encoding were considered for this study, one-hot encoding and embedding. Our results suggest that embedding is an optimal approach, as it allows the model to learn representations similar to the amino acid features, which results in further improvements in the ability to identify putative sites of modification.

Furthermore, DeepSuccinylSite corroborates previous indications in the literature that have suggested a window size of 33 optimally reflects local chemical interactions in proteins that predict sites of PTM due to its performance in metrics like MCC. One of the important parameters was embedding dimension. DeepSuccinylSite was trained with different dimensions ranging from 9 to 33. With increase in dimension, training time also increased. Though there was not a significant difference between dimension sizes 15 and 21, considering the number of amino acid residues and slightly better result, 21 was chosen as the embedding dimension for this study. Finally, for window size 33 with embedding dimension 21, DeepSuccinylSite achieved efficiency scores of 0.79, 0.69 and 0.48 for sensitivity, specificity and MCC, respectively.

For further improvements, instead of current protein sequence-based window sequence, we can extract structure-based window sequence centered around the site of interest and use that window as the input. When the structure of the protein is not available, protein structure prediction pipelines like I-TASSER [27] or ROSETTA [28], can first be used to predict the structure. Since the structure of the proteins are more conserved than sequence, we hope to capture evolutionary information better and thus obtain better prediction accuracy. Moreover, we could also improve the performance of the approach by creating multiple models using sequence-based windows, structure-based windows, physiochemical properties and then utilize voting approaches. Lastly, multi-window input, as done in DeepPhos [16], using our encoding technique can improve the performance. However, more datasets are required for these schemes and once more experimental data becomes available, we could explore this in more detail. We also explored the effects of data size on prediction performance (Additional file 1: Table S4 and Additional file 1: Figure S2). These studies suggest that, initially, the performance of our model increases with the increasing data size before reaching a plateau. This is somewhat contrary to the general consensus in deep learning that performance keeps increasing with the data size according to a power law. However, with more experimental data likely to be available in the future, we could perform a more comprehensive study on how performance scales with increasing data size. Perhaps, this might also suggest that with increasing data we might have to develop more complex deep learning models.

Utilizing the unique architecture described in this paper, the DeepSuccinylSite model shows a substantial improvement in predictive quality over existing models. The utility of this model is in its ability to predict lysine residues that are likely to be succinylated. Accordingly, this model could be utilized to optimize workflows for experimental verification of succinylation sites. Specifically, use of this model could significantly reduce the time and cost of identification of these sites. This model may also have some utility in hypothesis generation when PTM presents itself as likely explanation for observed biological phenomenon.

## Conclusion

In this study, we describe the development of DeepSuccinylSite, a novel and effective deep learning architecture for the prediction of succinylation sites. The primary advantage of using this model over other machine learning architectures is the elimination of feature extraction. As a consequence, other PTM sites could be easily applied in this model. Since this model only utilizes two convolutional layer and one max-pooling layer to avoid overfitting for the current data, provision of new data sources may allow for further modification of this model in the future. In conclusion, DeepSuccinylSite is an effective deep learning architecture with best-in-class results for prediction of succinylation sites and potential for use in general PTM prediction problems.

## Supplementary information

**Additional file 1: **Contains supplementary tables and figures referred to in the text. We describe various other deep learning architectures, other machine learning architectures, cross-validation results and independent test results for different sample sizes. **Table S1.** Independent Test Results. **Table S2.** Independent test result for different machine learning architectures. **Figure S1.** ROC curve for feature based DL-model. **Table S3.** Cross-validation (CV) results for different run. **Table S4.** Independent test results for different sample sizes. **Figure S2.** MCC and AUC for independent test for different sample sizes.

## Data Availability

The datasets and models analyzed during the current study along with the supplementary materials are available in https://github.com/dukkakc/DeepSuccinylSite.

## Abbreviations

AUC: Area under ROC curve
CNN: Convolutional Neural Network
DL: Deep learning
LSTM: Long short-term memory
MCC: Mathew correlation coefficient
PTM: Post translational modification
ReLU: Rectified linear unit
RNN: Recurrent neural network
ROC: Receiver operator characteristics

## References

[1] Hasan MM, Khatun MS. Prediction of protein Post-Translational Modification sites: An overview. Ann Proteom Bioinform. 2018;2:049-57. 10.29328/journal.apb.1001005.

[2] Medzihradszky KF (2005). Peptide sequence analysis. Methods Enzymol.

[3] Agarwal KL, Kenner GW, Sheppard RC (1969). Feline gastrin. An example of peptide sequence analysis by mass spectrometry. J Am Chem Soc.

[4] Welsch DJ, Nelsestuen GL (1988). Amino-terminal alanine functions in a calcium-specific process essential for membrane binding by prothrombin fragment 1. Biochemistry.

[5] Slade DJ, Subramanian V, Fuhrmann J, Thompson PR (2014). Chemical and biological methods to detect post-translational modifications of arginine. Biopolymers.

[6] Umlauf D, Goto Y, Feil R (2004). Site-specific analysis of histone methylation and acetylation. Methods Mol Biol.

[7] Jaffrey SR, Erdjument-Bromage H, Ferris CD, Tempst P, Snyder SH (2001). Protein S-nitrosylation: a physiological signal for neuronal nitric oxide. Nat Cell Biol.

[8] Xu Y, Ding YX, Ding J, Lei YH, Wu LY, Deng NY (2015). iSuc-PseAAC: predicting lysine succinylation in proteins by incorporating peptide position-specific propensity. Sci Rep.

[9] Jia J, Liu Z, Xiao X, Liu B, Chou KC (2016). iSuc-PseOpt: identifying lysine succinylation sites in proteins by incorporating sequence-coupling effects into pseudo components and optimizing imbalanced training dataset. Anal Biochem.

[10] Jia J, Liu Z, Xiao X, Liu B, Chou KC (2016). pSuc-Lys: predict lysine succinylation sites in proteins with PseAAC and ensemble random forest approach. J Theor Biol.

[11] Hasan MM, Yang S, Zhou Y, Mollah MNH (2016). SuccinSite: a computational tool for the prediction of protein succinylation sites by exploiting the amino acid patterns and properties. Mol BioSyst.

[12] Hasan MM, Khatun MS, Mollah MNH, Yong C, Guo D (2017). A systematic identification of species-specific protein succinylation sites using joint element features information. Int J Nanomedicine.

[13] Hasan MM, Kurata H (2018). GPSuc: global prediction of generic and species-specific Succinylation sites by aggregating multiple sequence features. PLoS One.

[14] Ning Q, Zhao X, Bao L, Ma Z, Zhao X (2018). Detecting Succinylation sites from protein sequences using ensemble support vector machine. BMC Bioinformatics.

[15] Wang D, Zeng S, Xu C, Qiu W, Liang Y, Joshi T (2017). MusiteDeep: a deep-learning framework for general and kinase-specific phosphorylation site prediction. Bioinformatics.

[16] Fenglin Luo, Minghui Wang, Yu Liu, Xing-Ming Zhao, Ao Li. DeepPhos: prediction of protein phosphorylation sites with deep learning, Bioinformatics. 2019;35(16):2766–73. 10.1093/bioinformatics/bty1051 PMC6691328 30601936

[17] Fu H, Yang Y, Wang X, Wang H, Xu Y (2019). DeepUbi: a deep learning framework for prediction of ubiquitination sites in proteins. BMC Bioinformatics.

[18] Wu M, Yang Y, Wang H, Xu Y (2019). A deep learning method to more accurately recall known lysine acetylation sites. BMC Bioinformatics.

[19] LeCun Y, Bengio Y, Hinton G (2015). Deep learning. Nature.

[20] Chollet F, et al. Keras; 2015. https://keras.io.

[21] Bengio Y, Ejean Ducharme R, Vincent P, De Recherche Mathematiques C, D’Informatique Et Recherche Operationnelle D (2001). A Neural Probabilistic Language Model.

[22] Kulmanov M, Khan MA, Hoehndorf R (2017). DeepGO: predicting protein functions from sequence and interactions using a deep ontology-aware classifier. Bioinformatics.

[23] Kingma DP, Adam BJ (2014). A Method for Stochastic Optimization. arXiv e-prints [Internet].

[24] Kiefer J, Wolfowitz J (1952). Stochastic estimation of the maximum of a regression function. Ann Math Stat.

[25] Jain LC, Medsker LR. Recurrent neural networks: design and applications: CRC press, Inc.; 1999. 416 p.

[26] Hochreiter S (1997). #252, Schmidhuber r. long short-term memory. Neural Comput.

[27] Roy A, Kucukural A, Zhang Y (2010). I-TASSER: a unified platform for automated protein structure and function prediction. Nat Protoc.

[28] DiMaio F, Leaver-Fay A, Bradley P, Baker D, Andre I (2011). Modeling symmetric macromolecular structures in Rosetta3. PLoS One.

