# Correction: DeepSuccinylSite: a deep learning based approach for protein succinylation site prediction

**DOI:** 10.1186/s12859-022-04844-2

**Published:** 2022-08-22

**Authors:** Niraj Thapa, Meenal Chaudhari, Sean McManus, Kaushik Roy, Robert H. Newman, Hiroto Saigo, Dukka B. KC

**Affiliations:** 1 Department of Computational Science and Engineering, North Carolina A&T State University, Greensboro, NC USA; 2 Department of Computer Science, North Carolina A&T State University, Greensboro, NC USA; 3 Department of Biology, North Carolina A&T State University, Greensboro, NC USA; 4 Faculty of Information Science and Electrical Engineering, Kyushu University, Fukuoka, Japan; 5 Electrical Engineering and Computer Science Department, Wichita State University, Wichita, KS USA

## Correction: BMC Bioinformatics 2020, 21(Suppl 3):63 https://doi.org/10.1186/s12859-020-3342-z

After publication of this supplement article [1], it is requested to correct the below errors in the article:

On page 1, the Result of Abstract should be changed to:

Results: Using an independent test set of experimentally identified succinylation sites, our method achieved efficiency scores of 79%, 68.7% and 0.27 for sensitivity, specificity and MCC respectively, with an area under the receiver operator characteristic (ROC) curve of 0.8. In side-by-side comparisons with previously described succinylation site predictors, DeepSuccinylSite produces similar or better results compared to the other state-of-the-art predictors.

On page 7, Last paragraph on right should be changed from

Consequently, DeepSuccinylSite achieved a significantly higher performance as measured by MCC. Indeed, DeepSuccinylSite exhibited an ~ 62% increase in MCC when compared to the next highest method, GPSuc.

to:

Consequently, DeepSuccinylSite achieved an MCC score (at decision boundary of 0.5) on par with the top performingmethod, GPSuc.

On page 2, in Table 1, the negative data of Independent Test should be 2977 rather than 254.

On page 8, in Table 6, the MCC data of DeepSuccinylSite should be 0.27 rather than 0.48.
