# Supplementary material for: DeepSuccinylSite: a deep learning based approach for protein succinylation site prediction
Source: BMC Bioinformatics. 2020 Apr 23;21(Suppl 3):63. doi: 10.1186/s12859-020-3342-z (PMC7178942; doi:10.1186/s12859-020-3342-z)
Supplement: Supplementary file 1 — Additional file 1: Contains supplementary tables and figures referred to in the text. We describe various other deep learning architectures, other machine learning architectures, cross-validation results and independent test results for different sample sizes. Table S1. Independent Test Results. Table S2. Independent test result for different machine learning architectures. Figure S1. ROC curve for feature based DL-model. Table S3. Cross-validation (CV) results for different run. Table S4. Independent test results for different sample sizes. Figure S2. MCC and AUC for independent test for different sample sizes. [file 12859_2020_3342_MOESM1_ESM.docx]

**DeepSuccinylSite: a deep learning based approach for protein succinylation site prediction - Supplementary Materials: Additional file 1**

**Niraj Thapa, Meenal Chaudhari, Sean McManus, Kaushik Roy, Robert H. Newman, Hiroto Saigo, Dukka B. KC**

**1. Details of compared deep learning architecture using one hot encoding features**

The embedding deep learning architecture is compared with following models. The same training and testing dataset were used for the comparison. One hot encoding features were generated for the training and test data and fed to the input layer. Following are the one hot encoding architecture that we used for comparison. All models are implemented on Keras library with the Tensorflow backend.

**Model 1: Long Short Term Memory (LSTM) architecture:** The model contains four layers: input layer, LSTM layer, fully connected later, and an output layer. We used binary cross entropy as the loss function and Adam optimizer.

Input Layer: The protein sequences of window size 33 were one-hot encoded with the feature space of 21(added ‘-‘ for no sequence) providing a shape of (33,21).

LSTM layer: It uses 32 neurons, and the input shape of (33,21) as (timesteps, features).

Fully connected layer: A fully connected layer acts as a hidden layer with 16 parameters.

Output layer: Softmax activation was used and the number of output nodes is taken as 2 (i.e., succinylated or not).

**Model 2: RNN Architecture:** The model contains three layers with one hidden layer. We used mean squared error as the loss function and Adam optimizer.

Input layer: The protein sequences of window size 33 were one-hot encoded with the feature space of 21(added ‘-‘ for no sequence) providing a shape of (33,21).

RNN Layer: Simple RNN layer was used with 32 neurons, input shape as (timesteps, features) of (33,21) similar to the LSTM architecture. Activation function used was ‘ReLU’.

Fully connected layer: A hidden layer with 21 parameters was used.

Output layer: Softmax activation was used and the number of output nodes is taken as 2 (i.e., succinylated or not).

**Model 3: RNN-LSTM Architecture:** The model contains five layers. We used mean squared error as the loss function and Adam optimizer.

Input layer: The protein sequences of window size 33 were one-hot encoded with the feature space of 21(added ‘-‘ for no sequence) providing a shape of (33,21).

LSTM layer: It has 4 neurons, and the input shape of (33,21) as (timesteps, features) with stateful kept as False.

Fully connected layer: A hidden layer with 21 parameters was used.

RNN layer: A Simple RNN layer with 32 neurons and activation function is ReLU.

Output layer: Softmax activation was used and the number of output nodes is taken as 2 (i.e., succinylated or not).

**Model 4: Random Forest**

To compare deep learning architecture with traditional machine learning algorithm, we compared the results with a Random Forest-based model. Scikit-learn was used to implement random forest, feature reduction was done using the best features from the random forest classifier.

Results:

Table S1. Independent Test Results

| Model | MCC/25 | Sensitivity | Specificity | AUC |
| --- | --- | --- | --- | --- |
| LSTM | 0.36 | 0.74 | 0.66 | 0.68 |
| LSTM-RNN | 0.24 | 0.63 | 0.61 | 0.66 |
| RNN | 0.20 | 0.70 | 0.49 | 0.59 |
| RF | 0.29 | 0.74 | 0.62 | 0.72 |

**2. Different Machine Learning Models using sequence based features**

For comparisons with the results of feature-based machine learning algorithms, we generated physico-chemical-based features like, Pseudo Amino acid Composition (PAAC), k-Spaced Amino Acid Pairs (AAP) and Composition, Transition and Distribution (CTD) as well as autocorrelation features like Moreau-Broto autocorrelation (MBA) and Entropy Features, such as Shannon entropy (SE), Relative entropy (RE), and Information Gain (IG). These features have been found to be effective for prediction of other posttranslational modifications using various machine learning architectures [1, 2].

We excluded any sequences with ‘-‘, while calculating the features. We then used XGBoost to extract prominent features, which provided better accuracy and obtained a total of 160 features at threshold 0.00145 that were used with various machine learning architectures (Table S2). Since XGBoost, itself, can also be used as a classifier, we also evaluated its performance directly. Finally, we implemented another Deep Learning architecture where the input was other ‘physico-chemical’ features. The performance of this “feature-based” DL model (FB-DL), as well as that of the other machine-learning models after applying XGBoost for 160 features, is shown in Table S2.

Table S2. Independent test result for different machine learning architectures.

| Model | MCC | Sensitivity | Specificity | AUC |
| --- | --- | --- | --- | --- |
| Random Forest | 0.21 | 0.56 | 0.65 | 0.64 |
| XGBoost | 0.25 | 0.72 | 0.53 | 0.69 |
| SVM | 0.23 | 0.66 | 0.58 | 0.63 |
| K Nearest Neighbors | 0.16 | 0.67 | 0.50 | 0.61 |
| Naïve Bayes | 0.23 | 0.74 | 0.48 | 0.65 |
| FB-DL | 0.27 | 0.80 | 0.44 | 0.69 |
| DeepSuccinylSite | 0.48 | 0.79 | 0.69 | 0.80 |

Based on these analyses, the performance of traditional machine learning models is not as good as that of our DL model that utilizes features only (i.e., FB-DL). More importantly, our original DL model (i.e., DeepSuccinylSite), which does not utilize features other than the primary amino acid sequence, performed better than the feature-based DL model.


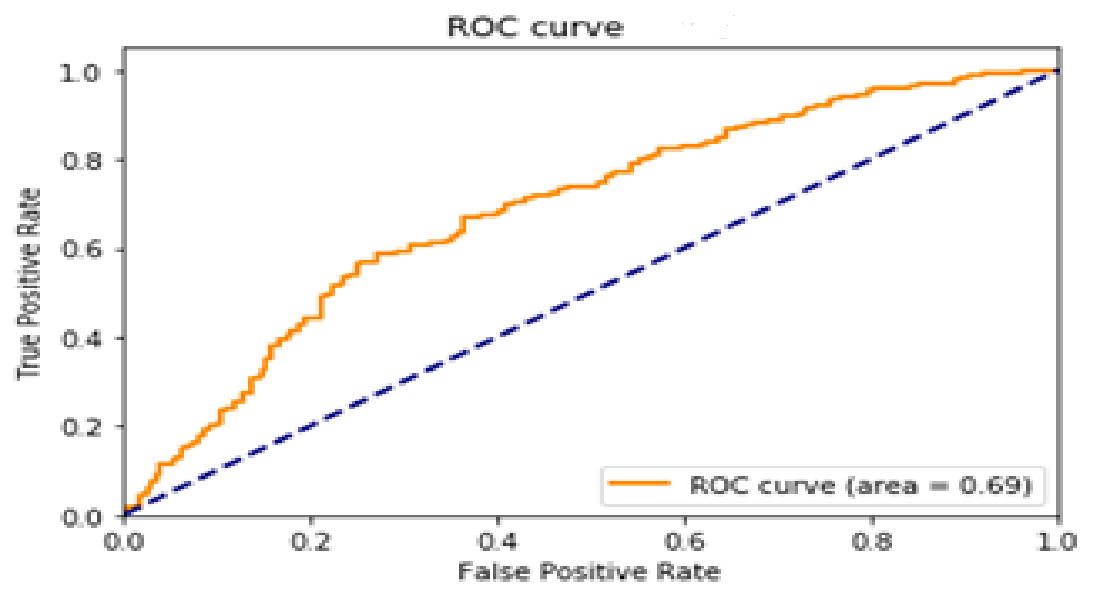


Figure S1: ROC curve for feature based DL-model

**3. DeepSuccinylSite: 10-fold cross-validation results:**

To deduce the robustness of our model, we performed 10-fold cross-validation multiple times with different seeds for differing randomness. The results are shown in Table S3. It can be seen that the result is consistent, suggesting that our model is robust.

Table S3. Cross-validation (CV) results for different run.

|  | MCC | Sensitivity | Specificity | AUC |
| --- | --- | --- | --- | --- |
| CV-1 | 0.516 +/- 0.0272 | 0.802 | 0.709 | 0.823 |
| CV-2 | 0.521 +/- 0.0274 | 0.792 | 0.728 | 0.820 |
| CV-3 | 0.516 +/- 0.0219 | 0.813 | 0.698 | 0.823 |
| CV-4 | 0.524 +/- 0.0200 | 0.802 | 0.720 | 0.824 |
| CV-5 | 0.529 +/- 0.0160 | 0.822 | 0.703 | 0.826 |

**4. DeepSuccinylSite: different data size comparisons:**

To see the effects of data size on prediction performance of the model, we trained our model on different sample sizes and performed the independent test (Table S4). These data suggest that performance metrics such as MCC and AUC increase with increasing sample size as we move from relatively small data sizes to larger ones; however, the performance gains appear to plateau at very larger data sizes (Table S4, Fig. S2). This suggests that, at least for this application, if more data becomes available in the future, we might have to explore more complex models to achieve better performance.

Table S4. Independent test results for different sample sizes.

| Sample Size | MCC | Sensitivity | Specificity | AUC |
| --- | --- | --- | --- | --- |
| 1000 | 0.14 | 0.48 | 0.66 | 0.60 |
| 3000 | 0.3 | 0.67 | 0.63 | 0.72 |
| 5000 | 0.40 | 0.72 | 0.68 | 0.77 |
| 7000 | 0.43 | 0.72 | 0.71 | 0.79 |
| 9500 (All) | **0.48** | 0.79 | 0.69 | 0.80 |

Figure S2. MCC and AUC for independent test for different sample sizes.

REFERENCES

1. Al-barakati HJ, McConnell EW, Hicks LM, Poole LB, Newman RH, Kc DB. SVM-SulfoSite: A support vector machine based predictor for sulfenylation sites. Scientific Reports. 2018;8(1):11288.

2. Ismail HD, Jones A, Kim JH, Newman RH, KC DB. RF-Phos: A Novel General Phosphorylation Site Prediction Tool Based on Random Forest. BioMed Research International. 2016;2016:12.
